# Meta-Analysis of Failure of Prehospital Endotracheal Intubation in Pediatric Patients

**DOI:** 10.1155/2020/7012508

**Published:** 2020-05-02

**Authors:** Jhon Jairo Rodríguez, Luis Felipe Higuita-Gutiérrez, Edwar Arturo Carrillo Garcia, Esneider Castaño Betancur, Mauricio Luna Londoño, Sara Restrepo Vargas

**Affiliations:** ^1^Infettare Research Group, School of Medicine, Universidad Cooperativa de Colombia, Clínica Antioquia, IPS Universitaria Universidad de Antioquia, Medellín, Colombia; ^2^Infettare Research Group, School of Medicine, Universidad Cooperativa de Colombia, School of Microbiology, Universidad de Antioquia, Medellín, Colombia; ^3^Infettare Research Group, Universidad Cooperativa de Colombia, Medellín, Colombia

## Abstract

Prehospital care is essential for airway preservation in pediatric patients who require early endotracheal intubation to improve oxygenation and prevent aspiration. However, high frequencies of failure of endotracheal intubation have been reported for this age group. We aimed to analyze the frequency of failure of endotracheal intubation in pediatric patients within a prehospital context and compare it with adult patients. Thus, a systematic revision of literature with a meta-analysis was performed using a study search and selection strategy ensuring extensiveness, sensitivity, and reproducibility. Meta-analyses were performed for odds ratio, DerSimonian and Laird's *Q* test was used to assess heterogeneity, and Egger and Begg's test was used to assess publication bias. Overall, 17 papers and 8772 patients were included, and the main cause of prehospital care was assessed to be trauma. Failed endotracheal intubation frequency was 0.4%–52.6% in pediatric patients. The most frequent complication was with esophageal intubation. Forest plot suggests that risk of failure during intubation of pediatric patients is 3.54 fold higher than that observed for adults. It was concluded that airway management in pediatric patients within a prehospital context is a challenge for prehospital care providers because it entails clear physiological and anatomical differences and a low frequency of exposure to this kind of events as opposed to adults. These differences support a widely higher risk of failure of intubation, suggesting the necessity of consistently trained prehospital care providers to ensure proficiency in technique as well as availability of the required equipment.

## 1. Introduction

Prehospital care is an essential medical service during urgency and emergency situations because early care at the place where the event occurs helps to decrease avoidable mortality, morbidity, and future disabilities [1–4]. Prehospital care is particularly important in the pediatric population because trauma is the most common cause of morbidity and mortality in this age group [5, 6], and endotracheal intubation is usually required in patients with severe traumatic injuries to improve oxygenation and prevent aspiration [7].

Furthermore, because pediatric patients have high oxygen consumption and a scarce oxygen reservoir, their apnea tolerance is low [8]. Former papers have documented that children who arrive at the hospital without a pulse and with apnea have a lower probability of survival, and if they do survive, there is a higher probability of neurological deficit [9].

In this context, appropriate airway management in pediatric patients is fundamental to reduce complications that increase morbidity and mortality in this population; therefore, proper knowledge of anatomy and physiology is required to follow a systematic and efficacious approach [10, 11]. Nowadays, there are different devices for the airway approach, such as bag valve mask, supraglottic devices, or endotracheal tubes, with orotracheal intubation (OTI) being the gold standard in airway management [12–15].

Nevertheless, OTI used for prehospital care of pediatric patients imposes additional challenges as opposed to adults. On one hand, pediatric patients' airway structures are smaller and more vulnerable, and desaturation develops more quickly during intubation attempts. Conversely, prehospital care providers often lack experience in pediatric airway management and do not have many opportunities to intubate in field, besides the influence of factors related to availability and the correct selection of the devices to be used [15–17].

The characteristics of pediatric patients increase the occurrence of failed intubation; however, previous research is widely contradictory when measuring the frequency of this phenomenon. Cooper and Demaret et al. report a frequency of 0.4% and 7.6%, respectively, whereas Hawkes and Boswell et al. report a frequency of 39% and 52.6%, respectively [18–21]. Similarly, discrepancies are found when comparing failed intubation in pediatric patients versus adult patients with odds ratio ranging from 12.7, determined by Bankole et al. [22], and 1.8, determined by Demaret et al. [19]. This shows the need to systematize available data to summarize information about airway management within a prehospital context deriving in strategic plans aimed at reducing morbidity and mortality caused by these factors in pediatric patients.

Taking this into consideration, this study was designed to analyze the frequency of failed endotracheal intubation in pediatric patients within a prehospital context and compare it with adults, based on papers found in the literature.

## 2. Methodology

### 2.1. Type of Study

A systematic revision of literature with meta-analysis was performed following the identification, screening, selection, and inclusion stages (Preferred Reporting Items for Systematic Reviews and Meta-analyses).

### 2.2. Identification

The terms “endotracheal intubation,” “pediatric,” and “prehospital” were used limited to title/summary in Pubmed, Scielo, Scopus, Cochrane Library, and Google Scholar databases. Some of the algorithms used were as follows: ((endotracheal intubation [Title/Abstract]) AND pediatric [Title/Abstract]) AND prehospital [Title/Abstract] (ab: (endotracheal intubation)) AND (ab: (pediatric)) AND (ab: (prehospital)) (TITLE-ABS-KEY (endotracheal AND intubation) AND TITLE-ABS-KEY (pediatric) AND TITLE-ABS-KEY (prehospital). Results were exported to a common source (Zotero reference manager), and this stage was closed after eliminating duplicates.

### 2.3. Screening

Identified papers were screened by reading the abstracts and applying the following inclusion criteria: (i) original papers, (ii) papers published in English, Spanish, or Portuguese, (iii) papers on endotracheal intubation, and (iv) papers not performed in simulated environments.

### 2.4. Selection

After completion of the screening phase, the following exclusion criteria were applied after fully reading the papers: (i) papers not fully available owing to database restrictions, (ii) papers including <20 patients, (iii) papers without specification of values used to estimate the frequency of intubation failure, (iv) papers performed with patients aged >18 years, and (v) papers on intubations performed within a hospital context.

### 2.5. Inclusion

An Excel database was created using all selected papers and the following variables: year of publication, place where the study was performed, number of patients included, clinical condition for which prehospital care was required, number of patients with endotracheal intubation, number of patients with failed endotracheal intubation, number of patients with esophageal intubation, and other reported complications.

### 2.6. Evaluation of Reproducibility and Methodological Quality

This database was filled in duplicates, and the Kappa index was calculated to ensure reproducibility of data collection. Two investigators applied protocol independently, and all discrepancies were solved by consensus and addressing a third party, thus ensuring reproducibility of data collection. To evaluate the methodological quality, the STROBE (Strengthening the Reporting of Observational studies in Epidemiology) guide was applied.

### 2.7. Analysis of Information

The frequency of failed endotracheal intubations was calculated with relative frequencies and a 95% confidence interval. An odds ratio (OR) meta-analysis was performed with papers reporting broken down frequencies of failed endotracheal intubation between pediatric and adult patients using a random effects model owing to the heterogeneity of the papers. The degree of heterogeneity was assessed using the DerSimonian and Laird's *Q* test (chi-squared) with a Galbraith plot. Publication bias was assessed through via a funnel plot and Begg statistical test. Forest plot was constructed as the overall result of meta-analysis to show differences between each study and the overall difference with their respective confidence intervals. Finally, a sensitivity analysis was performed to verify that the overall result is not influenced by a certain study. The analyses were performed using EPIDAT, version 3.1.

## 3. Results

**Figure 1:**
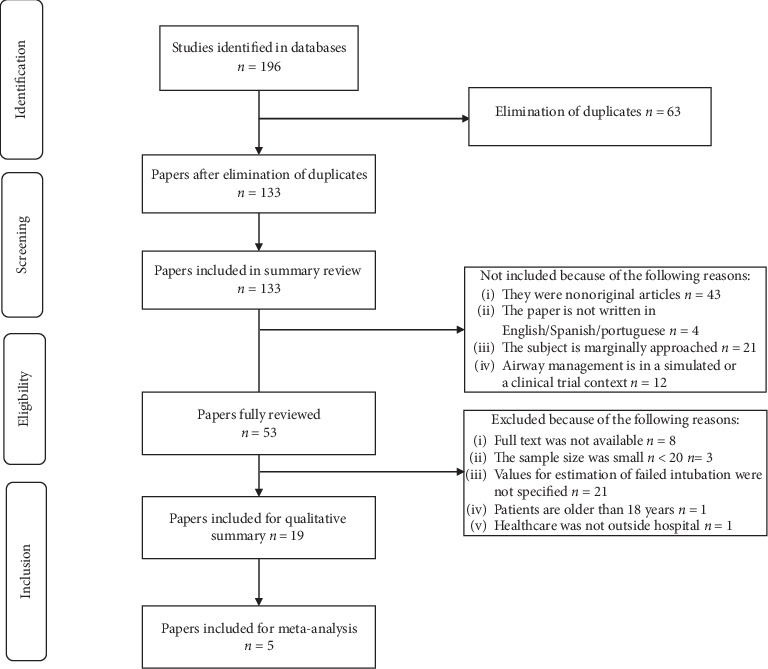
Flow diagram of study selection.

During early search, 196 papers were identified and 63 duplicates were eliminated. In total, 133 papers were screened after reading the abstracts, 80 of which were not included because of noncompliance with the inclusion criteria. During the selection stage, 53 papers were fully reviewed and 34 were excluded because of noncompliance with inclusion criteria (Figure 1)

In total, 19 papers were included in the qualitative summary, which were published between 1989 and 2018, and their studies were performed in different American states, followed by several European countries (Switzerland, Germany, and Belgium). In total, 8772 patients were included, and trauma was assessed to be the main cause of prehospital care, followed by cardiac arrest and seizures. The frequency of failed endotracheal intubation in pediatric patients ranged from 0.4% (CI 95% 0.1–1.5) to 52.6% (CI 95% 35.4–69.8) (Table 1).

**Figure 2:**
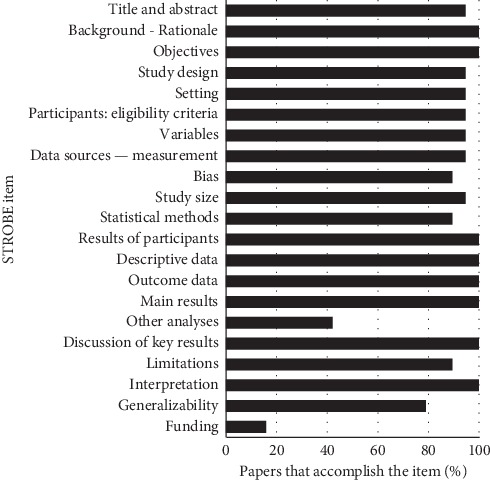
Evaluation of methodological quality.

An evaluation of the methodological quality of the incorporated papers exhibited that the percentage of compliance was greater than 80% of the criteria of the STROBE guide. The criteria that had the lowest agreement percentage were the explanation of other analysis and funding (Figure 2).

In 8 investigations, intubation-related complications were reported, the most frequent being esophageal intubation with values ranging from 0.9% (CI 95% 0.2–2.7) to 8.0% (CI 95% 1.0–26.0). Other complications included SpO_2_ < 90% after intubation, hypotension, aspiration, bleeding, bradycardia, trauma, sickness, and barotrauma (Table 2).

**Figure 3:**
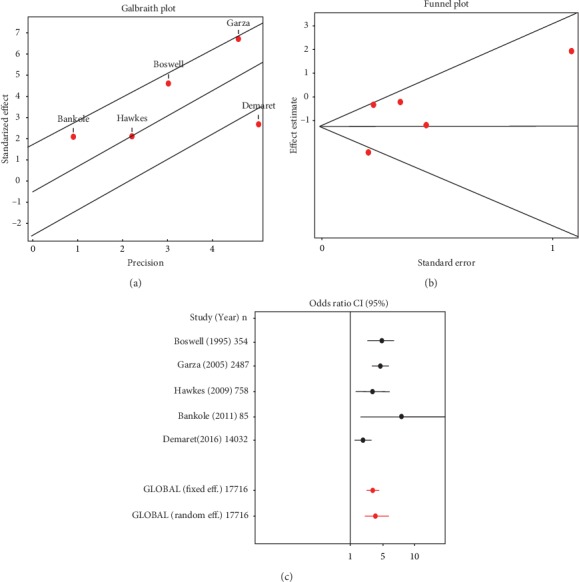
(a) Heterogeneity analysis using Galbraith plot, (b) publication bias by funnel plot, and (c) meta-analysis by forest plot.

An OR meta-analysis was performed based on the broken down frequency of failed endotracheal intubation in adult and pediatric patients. Both DerSimonian and Laird's *Q* test (*p* value 0.010) and Galbraith plot (Figure 3(a)) showed heterogeneity between papers; therefore, a random effects model was used. Begg statistical test (*p* value 0.9671) and funnel plot (Figure 3(b)) did not evidence publication bias.

The risk of failure of intubation in pediatric patients versus adult patients ranged from 1.83 (CI 95% 1.22–2.73) to 12.77 (CI 95% 1.54–106.05). The global measurement, based on the random effects model, suggests that the risk of intubation failure in pediatric patients is 3.54 fold (CI 95% 2.06–6.09) higher than that observed in adult patients (Table 3 and Figure 3(c)).

Based on the sensitivity analysis, the overall result did not seem to be significantly affected by individual papers because the conclusion does not change after excluding each study (Table 4).

## 4. Discussion

This study shows wide variability in the frequency of failed intubation in pediatric patients ranging from 0.4% (Cooper) [18] to 52.6% (Boswell) [21]. According to Tweed et al. [23], a main factor contributing to successful intubation by prehospital care providers is being consistently exposed to the performance of the procedure, both in the field and in simulated conditions, and this aspect is more relevant than the inherent risk factors of patients. In pediatric population, intubation requirements are approximately 4 per year, and in many places, training demands are minimal in terms of exposure, which may not be sufficient for prehospital staff to provide an adequate service when required [11, 15, 21, 23, 24]. Therefore, it is suggested that regardless of the demand, prehospital staff should be consistently trained in pediatric patient intubation procedures.

This study showed that the risk of intubation failure in pediatric patients is 3.54 fold (CI 95% 2.06–6.09) higher than that in adult patients, which is consistent with the previous publications [25, 26]. In this sense, it should be highlighted that pediatric patients have age-specific conditions which affect outcomes in airway management and impede an adequate visualization of the glottis, such as the proportion between head and body size, a higher and more anterior placement of the larynx, a larger tongue, tonsils and adenoid tissue, and a shorter neck and jaw. Conversely, a reduced functional residual capacity, besides increasing O_2_ consumption to 6/mL/kg/min as opposed to 3 mL/kg/min in adults and increasing CO_2_ production to 100–150 mL/kg/min as opposed to 60 mL/kg/min in adults, shortens the time for patients to present critical levels of these respiratory parameters, which can precipitate complications and collapse [27, 28].

Among the complications reported by papers, esophageal intubation was the most frequent one and was found in up to 8% of cases (Tarpgaard) [29]; it may arise from an inappropriate visualization of vocal folds because of the patient's conditions or because prehospital care providers in charge of airway management lacks experience in this field. Within this context, different techniques should be used to determine where the orotracheal tube should be placed correctly, including a physical examination where epigastric sounds are not heard during ventilation, a chest X-ray to visualize the orotracheal tube, capnography curve, expired CO_2_ value of >30 mm Hg, or steam water on the orotracheal tube walls. If any of these signs are altered, the risk of tube misplacement should be considered, which can cause additional complications to esophageal intubation such as bronchoaspiration, esophageal perforation, hypoxia, atelectasis, or even irreversible brain injury caused by hypoxia or death [30–32].

It should be highlighted that trauma is the most observed clinical condition in all papers included in this systematic review, which implies several particular characteristics of this patient type, possibly aggravating scenarios, and has a direct effect on outcomes. In this context, difficulties in airway approach related to physiological factors can be observed owing to injury mechanisms, which cause quick patient deterioration, as well as anatomical factors caused by the trauma itself, such as airway bleeding and anatomical integrity alterations [25, 32].

There are various devices and techniques for pediatric airway management which have been the object of debate over their utility, effectiveness, and long-term prognosis [15]. These techniques include bag-valve-mask ventilation (BVM), which is recommended by different authors [15], or supraglottic devices instead of OTI [12], regardless of the device used, which should be CO_2_ monitored at the end of expiration and/or capnography [24, 33, 34]. Despite all the available techniques, OTI remains the gold standard for airway management because its utility and safety mainly depend on nonpatient-related factors, such as consistent training of prehospital staff and sufficient pediatric intubation opportunities, which may reach only up to 4 or 5 per year, as stated previously [11, 15, 21, 23, 24].

Nowadays, there are 2 prehospital care models: the Anglo-American model, whose aim is for patients to arrive at the hospital and receive treatment there, and the French-German model, which focuses on the hospital arriving where the patient is. In the first model, early care is provided by technicians or paramedics and is eventually managed in the emergency center, whereas in the second model, early care is more complex and involves high technology equipment and physicians, mostly anesthesiologists, after which patients are transferred to specialized services according to the specific case [3, 35, 36]. The French-German model is widely criticized because physicians lack appropriate training on different scenarios and paramedics are not allowed to perform procedures without the presence of a physician. This may be fundamental when analyzing the results obtained.

Limitations of this study include variability of the included population, which affects result heterogeneity, as well as variables such as patient age or results analyzed according to age ranges, which may be important owing to the anatomical and physiological differences existing between age groups. Furthermore, characteristics of the prehospital care providers performing the intubation are not included and neither is their previous experience or education. Relevant information about causes, techniques, or relationships between complications and morbidity, mortality, and/or disability are not included either.

## 5. Conclusion

Airway management in pediatric patients within a prehospital context is a challenge for prehospital care providers because it implies clear physiological and anatomical differences as well as a low frequency of exposure to this type of events as opposed to adult patients. Because of such differences, the risk of intubation failure in pediatric patients is widely higher, which suggests the necessity of consistently trained prehospital care providers to ensure proficiency in technique, as well as availability of the required equipment. More studies should be conducted in this area to determine factors related to intubation failure and its effect on morbidity and mortality outcomes.

## Figures and Tables

**Table 1:** Frequency of failed endotracheal intubation in each study.

Author	Year	Place	Clinical condition	*n*	% (CI 95) intubation failure
Heschl et al. [16]	2018	Victoria, Australia	Trauma	87	1, 2 (0.03–6.24)
Tweed et al. [23]	2018	Texas, USA	Trauma, seizures, cardiac arrest, consciousness alterations, suffocation	27	51.8 (31.1–72.5)
Schmidt et al. [37]	2016	Zurich, Switzerland	Trauma, other medical conditions	215	1.9 (0.5–4.7)
Demaret et al. [19]	2016	Belgium	Trauma, cardiac arrest, others	353	7.7 (4.7–10.6)
Tarpgaard et al. [29]	2015	Central Denmark	Trauma, cardiac arrest, others	25	4.0 (0.1–20.3)
Hansen et al. [25]	2015	40 states, USA	Trauma, cardiac arrest, seizure	3124	19.0 (17.6–20.4)
Carlson et al. [38]	2015	33 states, USA	Trauma, breathing difficulty, cardiac arrest	3599	23.8 (22.4–25.2)
Bankole et al. [22]	2011	New Jersey, USA	Trauma	39	20.5 (6.6–34.5)
Hawkes [20]	2009	Denver, Colorado USA	Trauma, other medical conditions	23	39.1 (17.0–61.2)
Eich et al. [39]	2009	Göttingen, Germany	Trauma, seizures, SIDS, sepsis, toxicity, anaphylaxis, and others	58	1.7 (0.0–9.2)
Garza et al. [26]	2005	USA	Cardiac arrest	86	44.2 (33.1–55.3)
Ehrlich et al. [11]	2004	Virginia, USA	Trauma	59	16.9 (6.5–27.4)
Harrison et al. [40]	2004	Boston, USA	ND	143	4.9 (1.0–8.8)
Vilke et al. [41]	2002	San Diego, USA	Trauma, cardiorespiratory arrest, others	324	18.5 (14.1–22.9)
Cooper et al. [18]	2001	USA	Trauma	479	0.4 (0.1–1.5)
Boswell et al. [21]	1995	Georgia, USA	Trauma	38	52.6 (35.4–69.8)
Lavery et al. [42]	1992	New Jersey, USA	Trauma	30	20.0 (7.7–38.6)
Pointer [43]	1989	Alameda, USA	Trauma, drug overdose, others	36	11.1 (3.1–26.1)
Aijian et al. [44]	1989	California, USA	Cardiorespiratory arrest	28	35.7 (16.2–55.2)

SIDS: sudden infant death syndrome, ND: no data.

**Table 2:** Frequency of esophageal intubation and other procedure-related complications.

Author	% (CI 95) Esophageal intubation	Other complications
Tarpgaard et al. [29]	8.0 (1.0–26.0)	SpO2 < 90% after intubation, hypotension, aspiration
Hansen et al. [25]	1.2 (0.8–1.5)	Bleeding, bradycardia, hypotension, hypoxia, trauma, sickness
Bankole et al. [22]	2.6 (0.1–13.5)	ND
Eich et al. [39]	1.7 (0.0–9.2)	Misplaced tube
Ehrlich et al. [11]	3.4 (0.4–11.7)	Body intubation, aspiration, barotrauma, extubation
Vilke et al. [41]	0.9 (0.2–2.7)	ND
Pointer [43]	2.8 (0.1–14.5)	ND
Aijian et al. [44]	3.6 (0.1–18.3)	Misplaced tube

ND: no data.

**Table 3:** Odds ratio meta-analysis of failed endotracheal intubation in pediatric and adult patients.

Study	Year	*n*	OR	CI 95.0%
Boswell et al. [21]	1995	354	4.8457	2.4962–9.4068
Garza et al. [26]	2005	2487	4.593	2.9571–7.1339
Hawkes [20]	2009	758	3.0774	1.253–7.5584
Bankole et al. [22]	2011	85	12.7742	1.5387–106.0502
Demaret et al. [19]	2016	14032	1.8277	1.2239–2.7294
Fixed effects		17716	3.1133	2.4071–4.0267
Random effects		17716	3.5436	2.0611–6.0922

**Table 4:** Sensitivity analysis.

Omitted study	*n*	OR	CI 95.0%	Relative change %
Boswell et al. [21]	17362	3.2837	1.7015–6.3373	−7.33
Garza et al. [26]	15229	3.2651	1.669–6.3875	−7.86
Hawkes [20]	16958	3.7225	1.925–7.1985	5.05
Bankole et al. [22]	17631	3.2874	1.9038–5.6767	−7.23
Demaret et al. [19]	3684	4.5183	3.231–6.3185	27.51
GLOBAL	17716	3.5436	2.0611–6.0922	

## Data Availability

The processed data are available from the corresponding author upon request.
